# Erythropoietin Protects Against Cognitive Impairment and Hippocampal Neurodegeneration in Diabetic Mice

**DOI:** 10.3390/bs9010004

**Published:** 2018-12-28

**Authors:** Manal A. M. Othman, Ebrahim Rajab, Ahmed AlMubarak, Mohammed AlNaisar, Noora Bahzad, Amer Kamal

**Affiliations:** 1 Department of Anatomy, College of Medicine and Medical Sciences, Arabian Gulf University, 329 Manama, Bahrain; manalamo@agu.edu.bh; 2 Department of Histology, Faculty of Medicine, Assiut University, 71515 Assiut, Egypt; 3 School of Medicine, Royal College of Surgeons in Ireland, 228 Busaiteen, Bahrain; 4 Department of Physiology, College of Medicine and Medical Sciences, Arabian Gulf University, 329 Manama, Bahrain.; Ahmmed_ad@hotmail.com (A.A.); ameralansari@gmail.com (M.A.); noora.bahzad@gmail.com (N.B.); amerha@agu.edu.bh (A.K.)

**Keywords:** erythropoietin, diabetes, cognitive impairment, hippocampus, neurodegeneration

## Abstract

Administration of erythropoietin (EPO) is neuroprotective against a variety of experimentally-induced neurological disorders. The aim was to determine if EPO protects against hippocampal neurodegeneration as well as impairment of cognition and motor performance, associated with long-term diabetes. BALB/c mice were randomly allocated between control, diabetic and EPO-treated diabetic groups. EPO-treated diabetic mice were administered EPO 0.05 U/kg/day i.p. three times/week for 10 weeks. Cognition was assessed by Morris water maze. Brain samples were processed for light microscopic evaluation of hippocampus. Controls showed gradual improvement of cognitive performance in water maze when comparing latency (*p* < 0.01) and distance swum to reach the platform (*p* = 0.001). There was a similar trend for improvement in EPO-treated diabetics (*p* < 0.001). Latency did not improve in diabetic animals indicating lack of learning (*p* = 0.79). In probe trials, controls and EPO-treated diabetics spent more time in the training quadrant than expected by chance (*p* < 0.001). Diabetics did not show memory recall behavior; performance was significantly worse than expected by chance (*p* = 0.023). In diabetics, there was neurodegeneration in hippocampus and reduction in number of granule cells (*p* < 0.01) in the dentate gyrus. EPO treatment improved these neurodegenerative changes and preserved numbers of granule cells (*p* < 0.1, compared to controls). Erythropoietin treatment is protective against cognitive deficits and hippocampal neurodegeneration in diabetic mice.

## 1. Introduction

Dementia and Alzheimer’s disease (AD) are real consequences of uncontrolled diabetes [1]. Central insulin receptors are clustered in learning and memory areas of the brain such as the hippocampus [2]. Long-term diabetes is linked to impairment of synaptic plasticity, decreased dendritic complexity, decline of hippocampal neurogenesis, and hippocampal atrophy [3,4,5,6]. These changes are associated with moderate impairment of learning and memory in the middle-aged, and increased risk of dementia or AD in the elderly [7,8].

Erythropoietin (EPO) is a glycoprotein that is primarily formed in the kidney and is known for its major role in erythropoiesis [9]. Circulating EPO crosses the blood–brain barrier, and it is also produced and secreted in various parts of the central nervous system including the hippocampus, internal capsule, cortex, midbrain, cerebral endothelial cells, and astrocytes [10,11]. Erythropoietin receptors are expressed in the nervous tissue [12], and in normal mice hippocampal slices, EPO perfusion increases expression of long-term potentiation, which is a basic cellular model for learning and memory [13].

Exogenous administration of human recombinant EPO is neuroprotective against a variety of experimentally induced neurological disorders. These include, but are not limited to, ischemeic and traumatic brain injury, multiple sclerosis, Alzheimer’s disease, Parkinson’s disease, epileptic seizures, and diabetic neuropathy [10,14,15]. It is also associated with the prevention of cognitive impairment [15,16]. Erythropoietin protects cells of the nervous system by induction of neurogenesis and stimulation of angiogenesis, as well as being anti-excitotoxic, anti-apoptotic, anti-oxidative, anti-inflammatory, and exerting neurotrophic effects [17,18,19,20,21,22]. The mechanisms underlying these neuroprotective effects involve EPO binding to its receptor, with subsequent activation of phosphoinositide 3-kinase and protein kinase B (Akt) and signal transduction pathways including mTOR, Wnt, and WISP1, mammalian forkhead transcription factors of the O class, AMP kinase and silent mating type information regulation 2 homolog 1 [23].

Erythropoietin administration has the potential to treat various complications of diabetes including but not limited to fatigue, peripheral neuropathy, endothelial function and vascular perfusion [23,24,25,26,27]. Evidence of central neuroprotective effects per se in diabetic animals or human studies are limited. There is evidence of beneficial effects in a rat model of diabetes [28]. Also, a single dose of EPO has been shown to have beneficial effect on sustained attention/working memory in type 1 diabetic patients with hypoglycaemia [29]. The aim of this study was to investigate the potential preventative effects of chronic EPO treatment on spatial learning and memory as well as histology of the hippocampus in a mouse model of diabetes.

## 2. Materials and Methods

### 2.1. Animals

Male BALB/C mice (RRID: IMSR_CRL:28) 5–7 weeks old (20–25 g) were housed on sawdust and maintained on a 12 h light: 12 h dark cycle with free access to food and water. Mice were randomly allocated among three groups; this involved assigning n = 22 labelled cards to three groups (i.e., 7 × controls, 7 × diabetics and 8 × EPO-treated diabetics) and then drawing the cards blindly from a box, one at a time, for the allocation of each animal. We used only male mice in order to minimize any potential variability in the results due to sex differences. For induction of diabetes, mice were fasted for 4–6 h prior to daily intraperitoneal injection of 55 mg/kg STZ (S-0130, Sigma, Welwyn Garden, UK; dissolved in sodium citrate; pH 4.5) for a total of five days [30]. One week following the final dose of STZ, blood glucose was measured in a venous blood sample (obtained by tail prick) taken from each animal, using a strip-operated blood glucose monitor (OneTouch^®^ Ultra^®^ 2; Lifescan, Inc., Milpitas, CA, USA). In this method, streptozotocin-injected animals with a blood glucose of <15 mmol/L (280 mg/dL) were excluded from the study; however none of the STZ treated animals needed to be excluded. At the end of the study, blood glucose was measured in order to determine if there were any effects due to administration of EPO.

### 2.2. Protocol

EPO-treated diabetic mice were administered recombinant human EPO (E5627; Sigma-Aldrich, St. Louis, MO, USA) 0.05U/kg/day (dissolved in sodium citrate buffer; i.p.), and diabetic mice were administered an isovolume of sodium citrate buffer i.p., three times per week starting the day after the first i.p. administration of streptozotocin, and continuing for a period of ten weeks. Control mice were administered an isovolume of sodium citrate instead of the streptozotocin followed by three times per week sodium citrate buffer for the ten weeks. At the end of ten weeks treatment, learning and memory were assessed in the Morris water maze. Following this procedure, the mice were killed by overdosing with ether followed by decapitation, and the brains removed and processed for light microscopic evaluation of the hippocampus. This study was approved by the Research Ethics Committee of the Arabian Gulf University, Bahrain.

### 2.3. Morris Water Maze

Spatial learning and memory of mice were assessed daily for a total of 5 days in the Morris water maze [31,32,33]. Each animal underwent five acquisition trials per day for five consecutive days to learn the position of a hidden ‘escape’ platform, submerged 2 cm below the water surface, at a fixed location inside a circular swimming pool (140 cm diameter and 60 cm height, filled to a depth of 30 cm). At each trial, the mice were released from one of four predetermined positions on the perimeter of the pool. The starting position varied on each trial in a quasi-random sequence. Animals were given a maximum of two minutes to find the platform, and were allowed to remain on the platform for 30 s. Mice that failed to locate the platform were put onto it by the experimenter. The position and movement of the animals, in the pool, were captured and analyzed every 0.2 s, using a video-camera computer system, and ANY-maze video-tracking system (Stoelting Co, Wood Dale, IL, USA). Outcome measures were latency time and distance swum to reach the platform [34]. Speed of swimming which is a measure of motor function [35] was measured as control between the groups. Performance in the five daily trials was averaged to yield one data point per mouse per day. To measure any bias, a ‘probe trial’ was conducted on the sixth day. The platform was removed and each animal was allowed to swim for 60 s. In this probe trial, the selective search strategy was indicated if animals performed significantly above chance (25%).

### 2.4. Histology

After sacrificing the animals, the brains were obtained from the three assigned groups and processed for light microscopic evaluation of CA1, CA3, and DG regions of the hippocampus. The brains were fixed in 10% neutral buffered formalin, processed to prepare paraffin blocks, then a 5 µm horizontal sections [36], stained with Hematoxylin-Eosin (H&E) and cresyl violet stain (for Nissl granules). Processing and staining procedures were performed according to [37]. Slides were examined and photographs were obtained using a light microscope. For morphometric evaluation, the number of granule cells/field in the dentate gyrus (DG) region was counted in the three studied animal groups by a subject who was blinded to the experimental design. Counting of the nuclei of the cells was done viewing H&E stained sections at ×400 magnification in non-overlapping fields of each DG area in serial sections from the three experimental groups.

### 2.5. Data Analysis

Descriptive statistics including mean and standard deviation (SD) were used to summarize the data. Comparison between day 1 and day 5 measurements within groups were made using paired *t*-tests. For the water maze, between-subjects (between-group) and within-subject (in different time points) differences were tested using a two-way, factorial design repeated measures ANOVA with post hoc testing corrected for multiple hypothesis testing with Bonferroni correction (i.e., *p* value for significance set at 0.05/number of comparisons). For the probe trial, cell count data and blood glucose measurements, comparisons between groups were made using a one-way ANOVA and post-hoc *t*-test with Bonferroni correction for multiple comparisons. All statistical tests were performed blind to each group’s identification IBM SPSS Statistics for Windows version 22 (RRID:SCR_002865) (IBM Corp, Armonk, NY, USA). Statistical significance was set at a *p* value of less than 0.05.

## 3. Results

### 3.1. Water Maze

#### 3.1.1. Day 1

**Figure 1:**
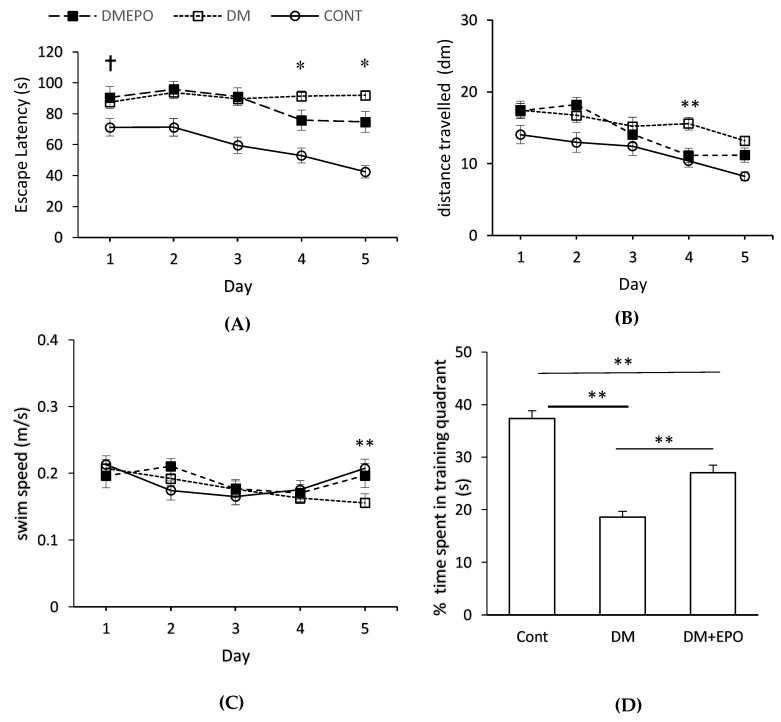
Morris water maze learning in control (Cont), diabetic (DM) and EPO-treated diabetic (DM+EPO) mice. Escape latency (**A**), distance swum to reach the platform (**B**) and swimming velocity (**C**) were measured across the 5 days of the trial. † Ten weeks of diabetes was associated with an increase in latency (*p* = 0.01) and distance swum (*p* = 0.02) by diabetic animals (with or without EPO supplementation) on day 1 compared to controls. * On day 4 and day 5, latency was longer in the diabetics compared to the EPO-treated diabetics. Distance swum was longer in the diabetics compared to the EPO-treated diabetics on day 4, but not different on day. Comparison of swimming speed among three groups indicated a marginally significant difference (*p* = 0.045) on day 5 due to a recovery in speed of control and EPO-treated diabetics, rather than a decrease in the speed of the diabetics (Figure 1C). In the probe (**D**), control and EPO-treated diabetics spent more time in the training quadrant than would be expected by chance (≤25%) (** *p* ≤ 0.01). Diabetic animals showed no apparent training behavior. EPO-treated diabetics spent less time in the training quadrant compared to controls (** *p* < 0.01). Diabetics spent less time in the training quadrant compared to EPO-treated diabetics (** *p* < 0.01). Data expressed as mean ± SEM.

Ten weeks of diabetes was associated with a reduction in cognitive performance (i.e., latency) compared to controls, at the start of the acquisition trials, (two-way (2-by-5) factorial repeated measures ANOVA, F = 3.229, degrees of freedom (d.f.) = 4, *p* = 0.014) (See Figure 1). In particular there was an increase in latency of diabetic animals (with or without EPO supplementation) on day 1 compared to controls (88.38 ± 3.67 versus 71.24 ± 5.51 s, *p* = 0.01). Similarly, there was an increase in distance swum by diabetics (with or without EPO supplementation) compared to controls on day 1 (17.43 ± 9.59 versus 14.04 ± 9.95 dm, *p* = 0.02). However, there was no difference in swimming speed between the groups (F = 0.319, degrees of freedom (d.f.) = 1, *p* = 0.57).

#### 3.1.2. Within Group Comparisons

Control animals showed improvements in their cognitive performance through days 1 to 5 when comparing latency (repeated measures ANOVA, F = 6.103, d.f. = 4, *p* < 0.001) and distance swum to reach the platform (repeated measures ANOVA, F = 5.869, d.f. = 4, *p* = 0.001) (Figure 1A,B). There was a similar trend for improvement in the EPO-treated diabetic animals (repeated measures ANOVA, latency: F = 2.996, d.f. = 4, *p* = 0.03; distance: F = 11.199, d.f. = 4, *p* < 0.001). However, latency did not change in the diabetic animals indicating a failure to improve in performance, across test days (repeated measures ANOVA, F = 0.432, d.f. = 4, *p* = 0.79), although there was a small reduction in distance swum (repeated measures ANOVA, F = 3.355, d.f. = 4, *p* = 0.01).

#### 3.1.3. Between Group Comparisons

On day 4 and day 5, latency was longer in the diabetics compared to the EPO-treated diabetics (day 4: 91.43 ± 33.72 s versus 75.87 ± 40.75 s; day 5: 91.90 ± 31.23 versus 74.77 ± 42.84 s) Distance swum was longer in the diabetics compared to the EPO-treated diabetics on day 4 (15.59 ± 8.92 versus 11.15 ± 6.25 dm), but not different on day 5 (13.20 ± 6.39 versus 11.20 ± 6.42 dm). When comparing these two variables across time and between different groups in a 3-by-5 factorial design, there was a significant difference in both latency (two-way ANOVA, F = 2.738, d.f. = 8, *p* = 0.005) and swimming distance (two-way ANOVA, F = 1.267, d.f = 8, *p* = 0.03). Comparison of swimming speed between three groups indicated that there was a marginally significant difference across factorial design (two-way ANOVA, F = 2.003, d.f. = 8, *p* = 0.045) on the account of day 5 (EPO-treated diabetics versus diabetic versus control on day 5, 0.197 ± 0.015 versus 0.156 ± 0.009 versus 0.207 ± 0.012 m/s). As can be seen, this difference in swimming speed on day 5 was due a recovery in swimming speed of control and EPO-treated diabetics compared to days 2–4, rather than a decrease in swimming speed of the diabetics (Figure 1C).

#### 3.1.4. Probe Trial

Control and EPO-treated diabetics spent more time in the training quadrant than would be expected by chance (≤25%) (one sample *t*-test: control 45 ± 2%, *t* (30) = 10.80, *p* = 3.72 × 10^−12^; EPO-treated diabetics, 32 ± 2%, *t* (30) = 4.31, *p* = 8.125 × 10^−5^) (Figure 1D). However, the diabetic animals showed no apparent memory recall behavior, rather their performance was significantly worse than would be expected by chance (one sample *t*-test; 22 ± 1%, *t* (30) = −2.09, *p* = 0.023). Comparing between the three groups, there was a significant difference in the percentage of time spent in the target quadrant (ANOVA, F (2, 90) = 47.45, *p* = 8.50 × 10^−15^). Controls spent more time in the training quadrant compared to EPO-treated diabetics (post hoc unpaired *t*-test: *t* (60) = 4.89, *p* = 7.84 × 10^−6^), and EPO-treated diabetics spent more time in the quadrant compared to diabetics (post-hoc unpaired *t*-test: *t* (60) = 4.70, *p* = 1.55 × 10^−5^).

### 3.2. Histology of the Hippocampus

#### 3.2.1. Hemotoxylin and Eosin Staining

**Figure 2:**
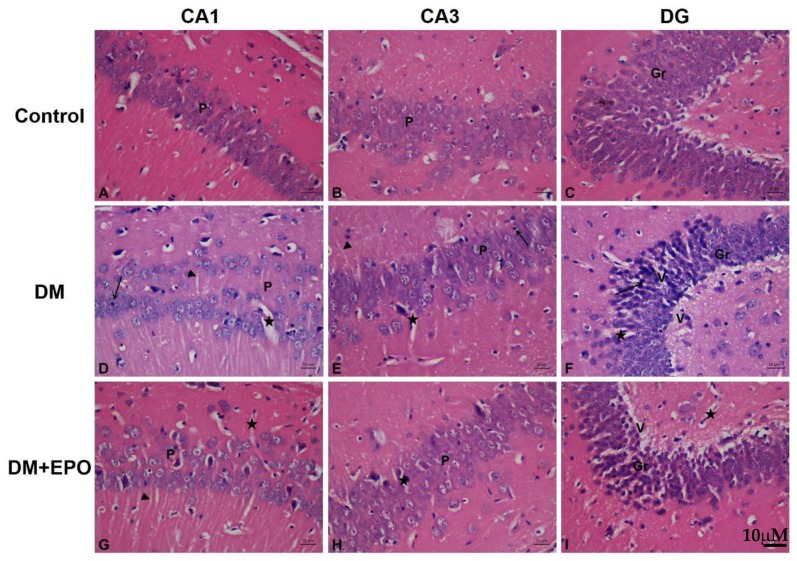
Hemeotoxylin and Eosin staining of the CA1, CA3 and dentate gyrus (DG) regions of the hippocampus from control, diabetic (DM) and erythropoietin-treated diabetic (DM+EPO) mice (images **A–I**). Magnification ×400. In the CA1 region of DM mice, there were areas of cellular loss and cells with dark small nuclei (arrow), note the wide blood capillaries (star) and clumping of cellular processes (arrow heads). In the CA3 region of DM mice there were numerous dilated blood capillaries (star), and dark cells with dark small nuclei (arrow), there was clumping of cellular processes (arrow head). In the DG of DM mice there was: marked cellular degeneration with many dark cells having small dark nuclei (arrow). There was wide blood capillaries (star) within the granular layer. There were areas of marked vacuolization in most areas (V). In the CA1 region of DM+EPO mice there was preservation of the pale cells and less dark cells. The widening of blood capillaries is still present (star). There was also persistence of clumping of cellular processes (star). In the CA1 region of DM+EPO mice there was preservation of the pale cells and less dark cells. The widening of blood capillaries is still present (arrow head). In the DG region of DM+EPO mice there was preservation of the pale cells and less dark cells. The vacuolization in some cells was still found (V).

In controls, the characteristic areas of the hippocampal formation were revealed as the hippocampus proper and the dentate gyrus. The CA1 (cornu amonis 1) region was formed of small pyramidal cells; CA3 (cornu amonis 3) was the region with larger less densely packed pyramidal cells. The dentate gyrus (DG) was observed as a coiled structure with an opened concave part directed to the hippocampus proper. The DG had the packed oval shaped granule cells. By contrast in diabetics, there were marked change in all regions of hippocampus in the form of disorganization and cell loss. Some cells had pale nuclei while others were dark. There was marked shrinkage in the size of the pyramidal cells in CA1 and CA3. In the DG the granule cells showed marked vacuolization with excess dark cells. Some cells showed signs of apoptosis in the form of shrinkage of the cytoplasm and darkening of both cytoplasm and nucleus. There was climbing of neuronal processes which appeared as whitish fibers. The blood capillaries appeared widened and congested. In EPO-treated diabetics, there was improvement in the form of preservation of small pyramidal cells, and cellular components, as well as a marked decrease in the apoptotic cells. Although there was marked improvement in the cells, there was persistence of climbing of neuronal processes and widening of the capillaries (see Figure 2).

#### 3.2.2. Cresyl Violet Stain

**Figure 3:**
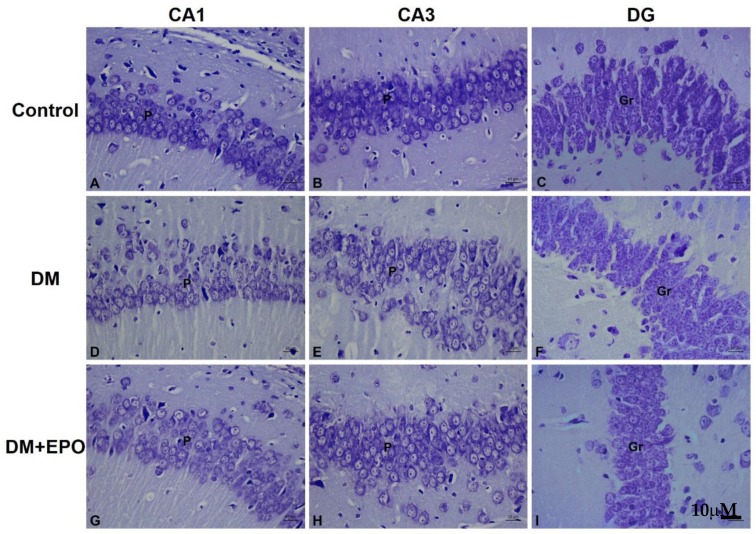
Cresyl violet staining of the CA1, CA3, and dentate gyrus (DG) regions of the hippocampus from control, diabetic (DM) and erythropoietin-treated diabetic (DM+EPO) mice (images **A**–**I**). Magnification ×400. Across all three regions in control mice, was observed Nissl granular constituents of the cytoplasm of cells which appeared dark violet, the nuclei of cells also appeared dark violet. P: pyramidal cells, Gr: granule cells. The rest of cytoplasm appeared lightly stained. In DM mice, Nissl granular components of the cytoplasm of cells were decreased compared to controls; granules in the cytoplasm of cells showed the cells paler in staining with less ribosomes inside. This was observed in all three regions. In DM+EPO mice, Nissl staining of the cells showing improvement in the Nissl granules and darker staining with more ribosomes inside compared to controls. This was in all three regions.

In controls, the cytoplasm of the cells was colored bright violet, and the Nissl substance (ribosomes) together with the nuclei were colored dark violet which was revealed as small dot like structures within the cytoplasm. By contrast, in diabetics, the cytoplasm of the cells appeared pale with less ribosomes compared to the control group. In EPO-treated diabetics, there was improvement in the cytoplasm of the cells as the cells appeared darker than the diabetes group with more ribosomes similar to that revealed in the control group (see Figure 3).

#### 3.2.3. Cell Count

**Figure 4:**
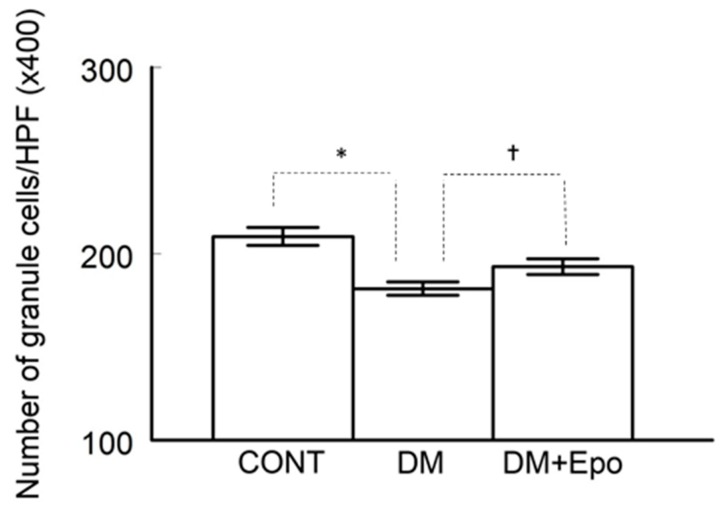
Number of granule cells/high power field (HPF) in the dentate gyrus (DG) was counted and compared among the control (CONT), diabetic (DM) and EPO-treated diabetics (DM+Epo). Counting cell nuclei was done viewing H&E stained sections at 400 magnification in non-overlapping fields of each DG area in serial sections from the three experimental groups. * Number of granule cells was lower in diabetics compared to control (*p* < 0.01). † Number of cells was lower in diabetics compared to EPO-treated diabetics (*p* < 0.01). Data expressed as mean ± SEM.

The number of granule cells in the dentate gyrus was decreased in diabetic groups, and marginally so in EPO-treated diabetics, compared to controls (ANOVA, F (2, 90) = 27.23, *p* = 5.70 × 10^−10^; unpaired, *t*-tests assuming unequal variance: control versus diabetes, *t* (54) = 6.73, *p* = 1.135 × 10^−8^; control versus EPO-treated diabetics, *t* (54) = 1.833, *p* = 0.072. In addition, there was a decrease in cell count of diabetics compared to EPO-treated diabetics (unpaired *t*-test: *t* (60) = −5.986, *p* = 1.294 × 10^−7^) (see Figure 4).

### 3.3. Blood Glucose

One week following administration of STZ, blood glucose confirmed induction of diabetes in the STZ-treated mice compared to controls (i.e., ≥15 mmol/L). Blood glucose of the diabetic groups was different from controls, both at baseline and at the end of the study (ANOVA: F (5, 30) = 143.23, *p* = 5.40 × 10^−20^; see Table 1). By the end of the study, blood glucose in the control animals had not changed compared to baseline (paired *t*-test: *t* (12) = 1.45, *p* = 0.173). In the two diabetic groups however, blood glucose increased significantly (see Table 1) and to a similar magnitude by the end of the study (unpaired *t*-test: *t* (12) = 1.05, *p* = 0.316). For both diabetic groups blood glucose remained significantly higher compared to controls at the end of the study (unpaired *t*-tests: control versus diabetes: *t* (12) = −30.24, *p* = 1.07 × 10^−12^; control versus EPO-treated diabetics: *t* (12) = −17.23, *p* = 7.875 × 10^−10^) (see Table 1).

## 4. Discussion

In this study, it has been shown that chronic EPO treatment is protective against cognitive deficits and hippocampal neurodegeneration, associated with STZ-induced diabetes in BALB/c mice. These beneficial effects of EPO treatment are not associated with improvements in blood glucose.

Data from as early as day 1 of the water maze test showed that 10 weeks of diabetes (with or without EPO treatment) resulted in impaired cognitive performance (i.e., escape latency and distance) compared to controls. Diabetes also impaired learning over the 5 day of the water maze test, indeed it worsened during the period of the test. Water-maze testing involves not only cognitive function of the brain; as target selection, selective attention and formation of external environment map in addition to memory formation and retrieval, but also on the status of the peripheral nervous system including the integrity of the sensorimotor connections and performance. Previously we have investigated the involvement of the sensorimotor system by testing a separate diabetic group of animals in the same water maze, but this time with making the platform visible [31]. In this case the animals will not need the hippocampus for spatial orientation or making memories about the location of the hidden disc. Those animals performed as good as the controls, which may suggest that although the sensorimotor deficit can affect theoretically the results of these animals but evidently these effects do not account fully for the actual deficits they had. In the current study, the deficit performance of the diabetic mice was not due to impaired motor performance based on the similarity in swimming speed between the groups. Differences in swimming speed on day 5 appear to be due to a recovery in the swimming speed of the control and EPO-treated diabetic rather than a reduction in speed of the diabetics. Furthermore, diabetic animals showed no apparent memory recall behavior in the probe trial, and their performance was significantly below the threshold for chance. These results broadly support previous animal studies of spatial learning and memory impairment associated with long-term severe diabetes [31,38,39]. Ten weeks of diabetes also resulted in marked cellular disruption across different areas of the hippocampus, in the form of deeply stained shrunken neuronal cells with deep pyknotic nuclei, which are suggestive of cell death [40,41,42]. In addition, there was a decrease in the number of granule cells in the dentate gyrus, and this finding is consistent with previous studies of experimentally induced diabetes [43,44,45]. Previous studies have suggested that this is a duration-related effect of diabetes on decline in hippocampal neuronal density, which takes more than 8 weeks to become apparent [44], and which may be a major contributing factor to impairments of memory and learning [45]. Central insulin receptors are clustered in learning and memory areas of the brain including the hippocampus [2], and insulin resistance is linked to disrupted energy metabolism, impaired mitochondrial function, oxidative stress and DNA damage ultimately leading to increased hippocampal atrophy and cognitive impairment [3]. It has been proposed that apoptosis occurs in diabetes due to increase in the expression of Bax proapoptotic gene and decrease the expression of Bcl-2 and Bcl-XL antiapoptotic genes as well as increasing caspase 3 activity [46]. Furthermore, elevated blood glucose and oxidative stress may increase leakiness of the blood brain barrier to harmful substances thereby exacerbating structural and functional damage, probably due to neuronal apoptosis [44,46].

Erythropietin-treated diabetics showed an improvement in cognition (i.e., escape latency and distance) on day 4 and escape latency on day 5 of the water maze test. In the probe trial, controls and EPO-treated diabetics spent more time in the training quadrant than would be expected by chance, unlike the diabetic animals which showed no apparent memory recall behavior; the effect of EPO was intermediate between the controls and untreated diabetics. Together, these findings suggest that EPO treatment improves spatial memory in long-term streptozotocin-induced diabetes. EPO-treated diabetics also showed improvements in the structure of different areas of the hippocampus, as well as increase in the number of granular cells in the dentate gyrus, compared to controls, suggesting a neuroprotective role. These findings, along with the lack of effect of EPO on blood glucose, support previous suggestions that EPO can have beneficial effects on memory in a rat model of diabetes, and type 1 diabetic patients without changing blood glucose [28,29]. More broadly, these neuroprotective effects are consistent with the findings of other rodent models of neuronal degeneration including sevofluorane-induced brain injury [47], transient middle cerebral artery occlusion [48], and Parkinson disease [49].

We utilized a multiple, low-dose STZ protocol for induction of diabetes [50]. However, blood glucose at the end of the study was higher than expected (Diabetic 31.6 ± 0.7mmol/L and Diabetic + EPO 30.1 ± 1.3 mmol/L) and more typical of severe diabetes with irreparable damage to the pancreatic β cells likely in spite of EPO administration [51,52]. This suggests that our colony of BALB/c mice were particularly sensitive to the STZ and may partly explain why unlike previous studies we did not see an expected improvement in blood glucose with EPO treatment [53,54]. Another reason why EPO did not have an effect on blood glucose may be due to the dose that we utilized (0.15 U/kg/week) which is much lower than similar studies by Katz et al. [53] and Chen et al. [54] who dosed ob/ob mice with 10,880 U/kg/week and STZ-diabetic rats with 900 U/kg/week respectively, to demonstrate blood glucose lowering effects. The dose we used is also much lower than that shown to improve memory in a rodent model of Alzheimer’s disease (5000 U/kg/day) [55]. The reason we used a lower dose was based on availability of EPO. Our latency and probe trial data shows that the dose was still sufficient to induce cognitive and neuronal changes Our low dose of EPO may also explain why we observed improvements on day 4 and 5 in the water maze test, rather than earlier, suggesting that the EPO-treated animals were still taking taking longer than controls to learn the location of the platform. The low dose of EPO also seems a more likely reason for the less robust latency responses compared to the diabetic animals, rather than the number of animals. Previous studies suggest that a sample size of 5–10 rats is typically required in order to determine differences in learning and memory between treatment groups in the Morris Water Maze [33,56,57]. Finally, direct comparison with human studies is difficult partly because we used the i.p. route but in humans i.v. or s.c. is the more typical approach to administration of EPO. Our dose of 0.15 U/kg/week is ~1000 fold lower than the mean s.c. dose typically used in humans (~175 U/kg/week) for increasing hemeoglobin levels in patients with end-stage renal disease and anaemia [58]. However, we did not measure hemeatocrit in this study.

The increased number of granule cells in the dentate gyrus of EPO-treated diabetics suggests that the compensatory mechanism by the progenitor cells replace degenerative neurons, occurs much faster than in the diabetics alone. The dentate gyrus of the hippocampus undergoes continuous neurogenesis throughout life [59]. Changes in neurogenesis alter a number of key functions in the hippocampus, such as learning and memory [60]. Hippocampal neurogenesis and neuroplasticity appears to be sensitive to many pathogenic and treatment factors that are associated with metabolic diseases including diabetes. A growing literature provides strong evidence that diabetes negatively affects the morphological integrity of the hippocampus, which may contribute to cognitive and mood symptoms in diabetes [61]. The neuro-restorative effects of EPO have been ascribed to direct effects on neural stem cells, in rodent models of ischemic and traumatic brain injury [62,63]. Accordingly, deletion of EPO receptor (EPOR) in the nervous system reduces the sizes of neural stem cell pool and impairs injury-induced adult neurogenesis [62,64]. The mechanisms underlying these neuroprotective effects involve EPO binding to the EPOR, and subsequent activation of signal transduction pathways including mTOR, Wnt, and WISP1, mammalian forkhead transcription factors of the O class, AMP kinase and silent mating type information regulation 2 homolog 1 [23]. These may be the mechanisms of action of EPO in diabetes, but it requires further investigation.

This study shows that 10 weeks of diabetes induced dilatation of blood capillaries, and this effect was augmented by EPO. Longer duration studies (i.e., 16 weeks) of streptozotocin-induced diabetes have shown have shown pathological changes in the blood vessels of the hippocampus including lumen stenosis, vessel wall collapse, endothelial cell swelling and shedding [65]. The association of diabetes with vascular-injury-related diseases such as vascular dementia, infarcts and hemorrhages [66] suggests disruption of blood-brain barrier integrity resulting in functional compromise; currently there is a lack of data in this field [67]. Endothelial cells express EPO receptor mRNA and protein [68]. Erythropoietin enhances vascular endothelial growth factor VEGF) secretion in neural progenitor cells via PI3K/Akt and ERK1/2 signaling pathways and upregulates VEGF receptor expression in cerebral endothelial cells [69]. Furthermore, EPO treatment of endothelial cell cultures stimulates Jak2, the production of matrix metalloproteinase 2 and the formation of vascular structures [70].

A limitation of our study is that we chose to use BALB/C mice due to their availability. However, these mice are known to be poor learners in tests requiring spatial abilities such as the Morris water maze. This has been attributed previously to a number of factors including their higher anxiety compared to other strains, hippocampal particularities, poor visual acuity and lighting conditions during the test [71,72,73]. However, the values we saw in our study for latency of control animals are similar to what has been reported previously for BALB/C mice [71]. Another limitation of our study is that we did not include an EPO-treated control group. In this regard, studies suggest that EPO drives proliferative and anti-apoptotic responses in the developing and adult brain, thereby promoting neuroprotection [74]. Previously, the relationship between dose of exogenously administered EPO, and how much crosses the blood-brain barrier, has been investigated in samples of cerebrospinal fluid measurements [75]. It was beyond the scope of our study to do so, but we acknowledge that such measurements would have been a useful addition considering EPO’s low blood–brain barrier penetration [76], and the low but multiple dosing protocol that we used. A further limitation of our study is the lack of investigation of synaptic plasticity in the hippocampus. Long-term diabetes in rodents leads to a deterioration in learning and memory as well as hippocampal long-term potentiation and depression, which are forms of synaptic plasticity [77,78]. However, we have demonstrated previously in mouse hippocampal slices, that EPO decreases the excitatory neurotransmitter release probability and increases the expression of long-term potentiation in the hippocampus [13].

## 5. Conclusions

Chronic EPO-treatment is protective against cognitive deficits and hippocampal neurodegeneration associated with 10-weeks of STZ-induced diabetes in BALB/c mice. These beneficial effects are not associated with changes in blood glucose. The mechanism of these neuroprotective effects are unclear and further studies are warranted with consideration given to synaptic plasticity and EPO receptor signaling cascades.

## Figures and Tables

**Table 1:** Blood glucose measurements.

Group	Blood Glucose Baseline (mmol/L)	Blood Glucose End (mmol/L)	Statistical Comparisons: Baseline vs. End
control	8.6 ± 0.5	7.8 ± 0.4	*p* = 0.1733, *t* = 1.4478
Diabetic	18.6 ± 0.7 **	31.6 ± 0.7 **	*p* = 1.945 × 10^−8^, *t* = −13.0162
Diabetic + EPO	17.9 ± 0.6 **	30.1 ± 1.3 **	*p* =1.5916 × 10^−6^, *t* = −8.6924

Note: Blood glucose measurements confirmed induction of diabetes in the STZ-treated mice compared to controls (i.e., >15 mmol/L). Blood glucose of the diabetic groups was different from controls, both at baseline and at the end of the study (ANOVA: F (5, 30) = 143.23, *p* = 5.40 × 10^−20^; ** unpaired *t*-tests control versus diabetic groups: *p* < 0.01). Data expressed as mean ± SEM.
